# Supplementary material for: Targeting Viral and Cellular Cysteine Proteases for Treatment of New Variants of SARS-CoV-2
Source: Viruses. 2024 Feb 22;16(3):338. doi: 10.3390/v16030338 (PMC10976049; doi:10.3390/v16030338)
Supplement: Supplementary file 1 [file viruses-16-00338-s001.zip › viruses-2807808-supplementary.pdf]

viruses

MDPI

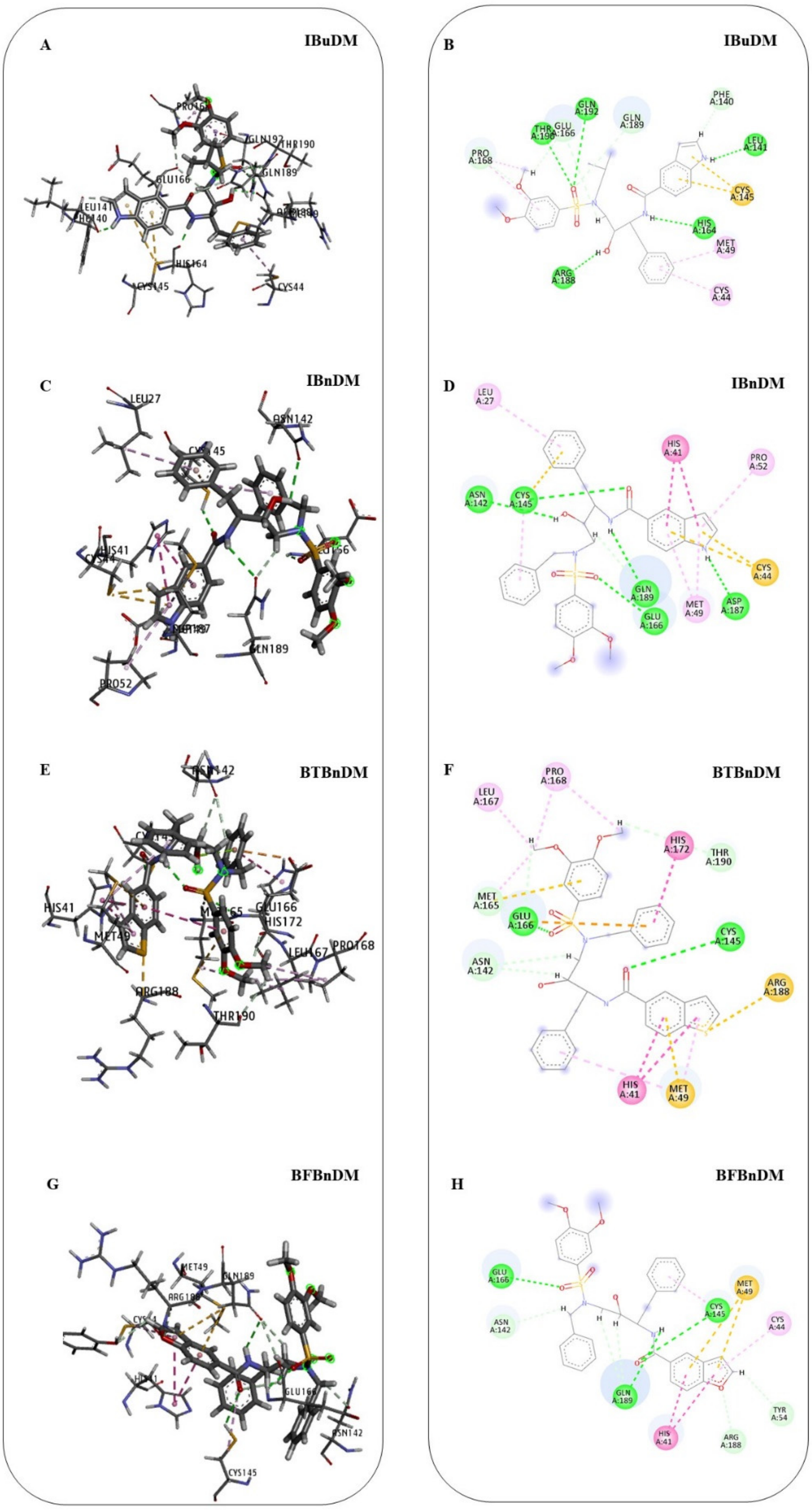

Figure S1. 3D interaction profile (left panel) and 2D interaction diagram (right panel) of the best-docked poses for 3CL^{pro}. Show 2D and 3D interactions of docked poses of IBuDM (A, B), IBnDM (C, D), BTBnDM (E, F), and BFBnDM (G, H) with the catalytic site residues of 3CL^{pro}.

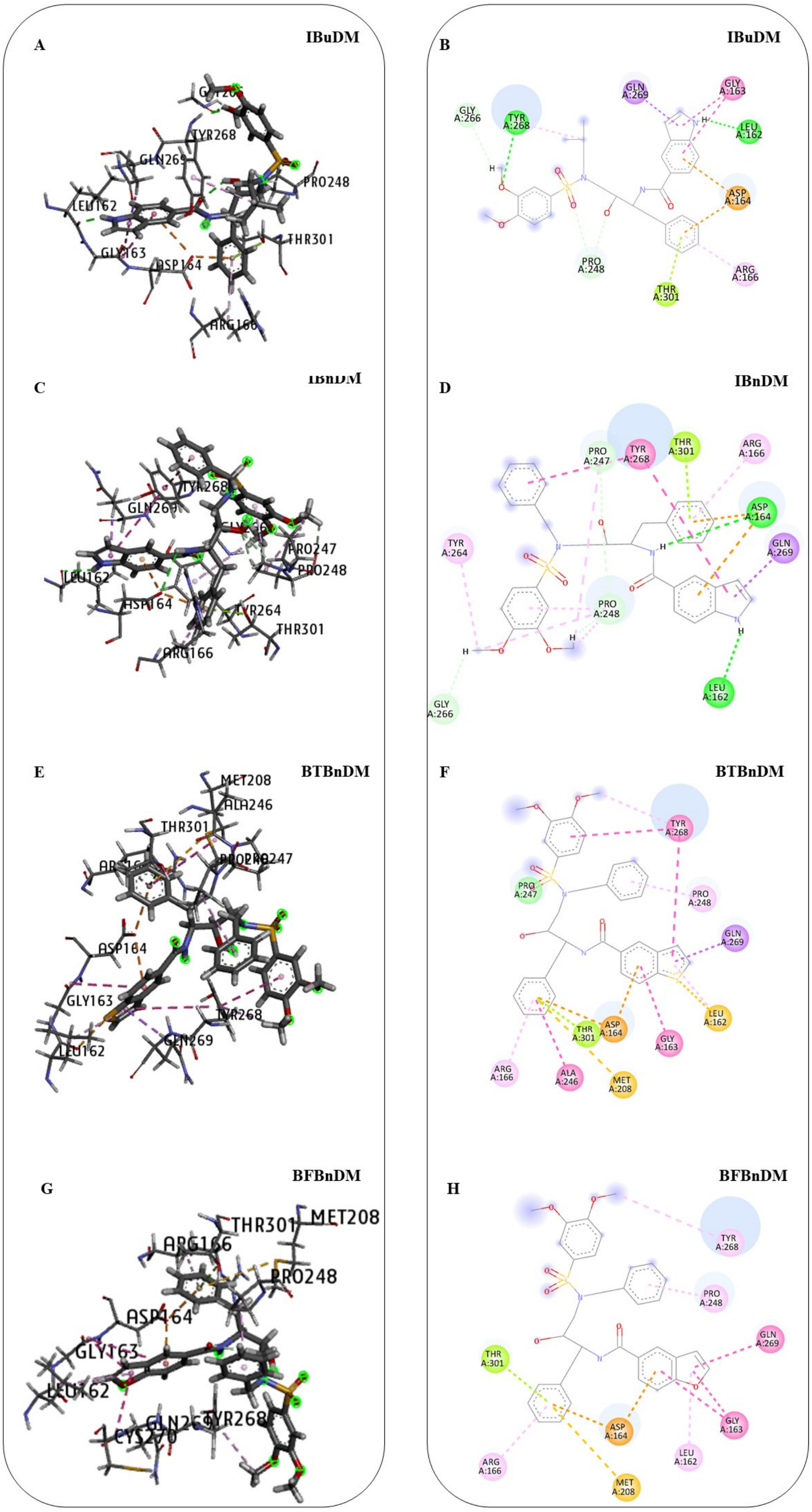

Figure S2. 3D interaction profile (left panel) and 2D interaction diagram (right panel) of the best-docked poses for PLpro. Show 2D and 3D interactions of docked poses of IBuDM (A, B), IBnDM (C, D), BTBnDM (E, F), and BFBnDM (G, H) with the catalytic site residues of PLpro.

Table S1. Dataset of HIV-1 inhibitors library.

Entry	Smiles	Energy of binding (kcal/mol) 3CL^{pro}
IBnDM	O[C@H](CN(CC1=CC=CC=C1)S(C2=CC(OC)=C(OC)C=C2)(=O)=O)C(CC3=CC=CC=C3)NC(C4=CC(C=CN5)=C5C=C4)=O	−7.1
BTBnDM	O[C@H](CN(CC1=CC=CC=C1)S(C2=CC(OC)=C(OC)C=C2)(=O)=O)C(CC3=CC=CC=C3)NC(C4=CC(C=CS5)=C5C=C4)=O	−6.9
IBuDM	O[C@H](CN(CC(C)C)S(C1=CC(OC)=C(OC)C=C1)(=O)=O)C(CC2=CC=CC=C2)NC(C3=CC(C=CN4)=C4C=C3)=O	−6.8
BFBnDM	O[C@H](CN(CC1=CC=CC=C1)S(C2=CC(OC)=C(OC)C=C2)(=O)=O)C(CC3=CC=CC=C3)NC(C4=CC(C=CO5)=C5C=C4)=O	−6.6
**1**	O[C@H](CN(CC(C)C)S(C1=CC=C(OC)C=C1)(=O)=O)CNC(OC2=CC(C=CS3)=C3C=C2)=O	> −6.5
**2**	O[C@H](CN(CC(C)C)S(C1=CC=C(OC)C=C1)(=O)=O)CNC(OC2=CC(C=CO3)=C3C=C2)=O	> −6.5
**3**	O[C@H](CN(CC(C)C)S(C1=CC=C(OC)C=C1)(=O)=O)C(CC2=CC=CC=C2)NC(OC3=CC(C=CN4)=C4C=C3)=O	> −6.5
**4**	O[C@H](CN(CC(C)C)S(C1=CC=C(OC)C=C1)(=O)=O)C(CC2=CC=CC=C2)NC(OC3=CC(C=CS4)=C4C=C3)=O	> −6.5
**5**	O[C@H](CN(CC(C)C)S(C1=CC=C(OC)C=C1)(=O)=O)C(CC2=CC=CC=C2)NC(OC3=CC(C=CO4)=C4C=C3)=O	> −6.5
**6**	O[C@H](CN(CC1=CC=CC=C1)S(C2=CC=C(OC)C=C2)(=O)=O)C(CC3=CC=CC=C3)NC(OC4=CC(C=CN5)=C5C=C4)=O	> −6.5
**7**	O[C@H](CN1[C@H](C(NC(C)(C)C)=O)CC(CCCC2)C2C1)COC(NC3=CC(C=CN4)=C4C=C3)=O	> −6.5
**8**	O[C@H](CN(CC(C)C)S(C1=CC(OC)=C(OC)C=C1)(=O)=O)COC(NC2=CC(C=CN3)=C3C=C2)=O	> −6.5
**9**	O[C@H](CN(CC(C)C)S(C1=CC=C([N+]([O-])=O)C=C1)(=O)=O)COC(NC2=CC(C=CN3)=C3C=C2)=O	> −6.5
**10**	O[C@H](CN1[C@H](C(NC(C)C)=O)CC(CCCC2)C2C1)COC(NC3=CC=CC4=C3C=CN4)=O	> −6.5
**11**	O[C@H](CN(CC(C)C)S(C1=CC(OC)=C(OC)C=C1)(=O)=O)COC(NC2=CC=CC3=C2C=CN3)=O	> −6.5
**12**	O[C@H](CN(CC(C)C)S(C1=CC=C([N+]([O-])=O)C=C1)(=O)=O)COC(NC2=CC=CC3=C2C=CN3)=O	> −6.5
**13**	O[C@H](CN(CC(C)C)S(C1=CC(OC)=C(OC)C=C1)(=O)=O)CNC(C2=CC(C=CN3)=C3C=C2)=O	> −6.5
**14**	O[C@H](CN(CC(C)C)S(C1=CC(OC)=C(OC)C=C1)(=O)=O)CNC(C2=CC(C=CS3)=C3C=C2)=O	> −6.5
**15**	O[C@H](CN(CC(C)C)S(C1=CC(OC)=C(OC)C=C1)(=O)=O)CNC(C2=CC(C=CO3)=C3C=C2)=O	> −6.5
**16**	O[C@H](CN(CC(C)C)S(C1=CC(OC)=C(OC)C=C1)(=O)=O)C(CC2=CC=CC=C2)NC(C3=CC(C=CS4)=C4C=C3)=O	> −6.5
**17**	O[C@H](CN(CC(C)C)S(C1=CC(OC)=C(OC)C=C1)(=O)=O)C(CC2=CC=CC=C2)NC(C3=CC(C=CO4)=C4C=C3)=O	> −6.5
**18**	O[C@H](CN(CC(C)C)S(C1=CC=C(OC)C=C1)(=O)=O)C(CC2=CC=CC=C2)NC(C3=CC(C=CN4)=C4C=C3)=O	> −6.5
**19**	O[C@H](CN(CC(C)C)S(C1=CC=C(OC)C=C1)(=O)=O)C(CC2=CC=CC=C2)NC(C3=CC(C=CS4)=C4C=C3)=O	> −6.5
**20**	O[C@H](CN(CC(C)C)S(C1=CC=C(OC)C=C1)(=O)=O)C(CC2=CC=CC=C2)NC(C3=CC(C=CO4)=C4C=C3)=O	> −6.5

**21**	O[C@H](CN(CC(C)C)S(C1=CC=C([N+]([O-])=O)C=C1)(=O)=O)C(CC2=CC=CC=C2)NC(C3=CC(C=CN4)=C4C=C3)=O	> −6.5
**22**	O[C@H](CN(CC(C)C)S(C1=CC=C([N+]([O-])=O)C=C1)(=O)=O)C(CC2=CC=CC=C2)NC(C3=CC(C=CS4)=C4C=C3)=O	> −6.5
**23**	O[C@H](CN(CC(C)C)S(C1=CC=C([N+]([O-])=O)C=C1)(=O)=O)C(CC2=CC=CC=C2)NC(C3=CC(C=CO4)=C4C=C3)=O	> −6.5
**24**	O[C@H](CN(CC(C)C)S(C1=CN=C([N+]([O-])=O)C=C1)(=O)=O)C(CC2=CC=CC=C2)NC(C3=CC(C=CO4)=C4C=C3)=O	> −6.5
**25**	CC(C)CN(S(C1=CC=C(OC)C=C1)(=O)=O)CC(O2)C(CC3=CC=CC=C3)NC2=O	> −6.5
**26**	O[C@H](CN(CC1=CC=CC=C1)S(C2=CC=C(OC)C=C2)(=O)=O)C(CC3=CC=CC=C3)NC(C4=CC(C=CN5)=C5C=C4)=O	> −6.5
**27**	O[C@H](CN(CC1=CC=CC=C1)S(C2=CC=C(OC)C=C2)(=O)=O)C(CC3=CC=CC=C3)NC(C4=CC(C=CS5)=C5C=C4)=O	> −6.5
**28**	O[C@H](CN(CC1=CC=CC=C1)S(C2=CC=C(OC)C=C2)(=O)=O)C(CC3=CC=CC=C3)NC(C4=CC(C=CO5)=C5C=C4)=O	> −6.5
**29**	O[C@H](CN(CC1=CC=CC=C1)S(C2=CC=C([N+]([O-])=O)C=C2)(=O)=O)C(CC3=CC=CC=C3)NC(C4=CC(C=CN5)=C5C=C4)=O	> −6.5
**30**	O[C@H](CN(CC1=CC=CC=C1)S(C2=CC=C([N+]([O-])=O)C=C2)(=O)=O)C(CC3=CC=CC=C3)NC(C4=CC(C=CS5)=C5C=C4)=O	> −6.5
**31**	O[C@H](CN(CC1=CC=CC=C1)S(C2=CC=C([N+]([O-])=O)C=C2)(=O)=O)C(CC3=CC=CC=C3)NC(C4=CC(C=CO5)=C5C=C4)=O	> −6.5

Table S2. Calculated free energies of binding (ΔG_B, in kcal/mol) of the selected compounds for 3CL^{pro} and PL^{pro}.

Compound	ΔG_B 3CL^{pro} PDB ID: 6LU7	ΔG_B PL^{pro} PDB ID: 7LBR
IBuDM	−6.6	−8.1
IBnDM	−7.1	−7.2
BTBnDM	−6.9	−7.8
BFBnDM	−6.8	−7.6

Table S3. Structures and calculated free energies of binding (ΔG_B, in kcal/mol) of the selected compounds for Cathepsin.

Compound	ΔG_B CatB PDB ID: 2IPP	ΔG_B CatL PDB ID: 5F02	ΔG_B CatK PDB ID: 5TUN	ΔG_B CatV PDB ID: 3H6S	ΔG_B CatS PDB ID: 6LU7
IBuDM	−9.5	−8.2	−8.1	−8.7	−9.7
IBnDM	−9.9	−8.6	−8.8	−9.1	−9.9
BTBnDM	−9.7	−8.3	−8.2	−8.8	−9.4
BFBnDM	−9.6	−8.5	−8.1	−8.5	−9.3

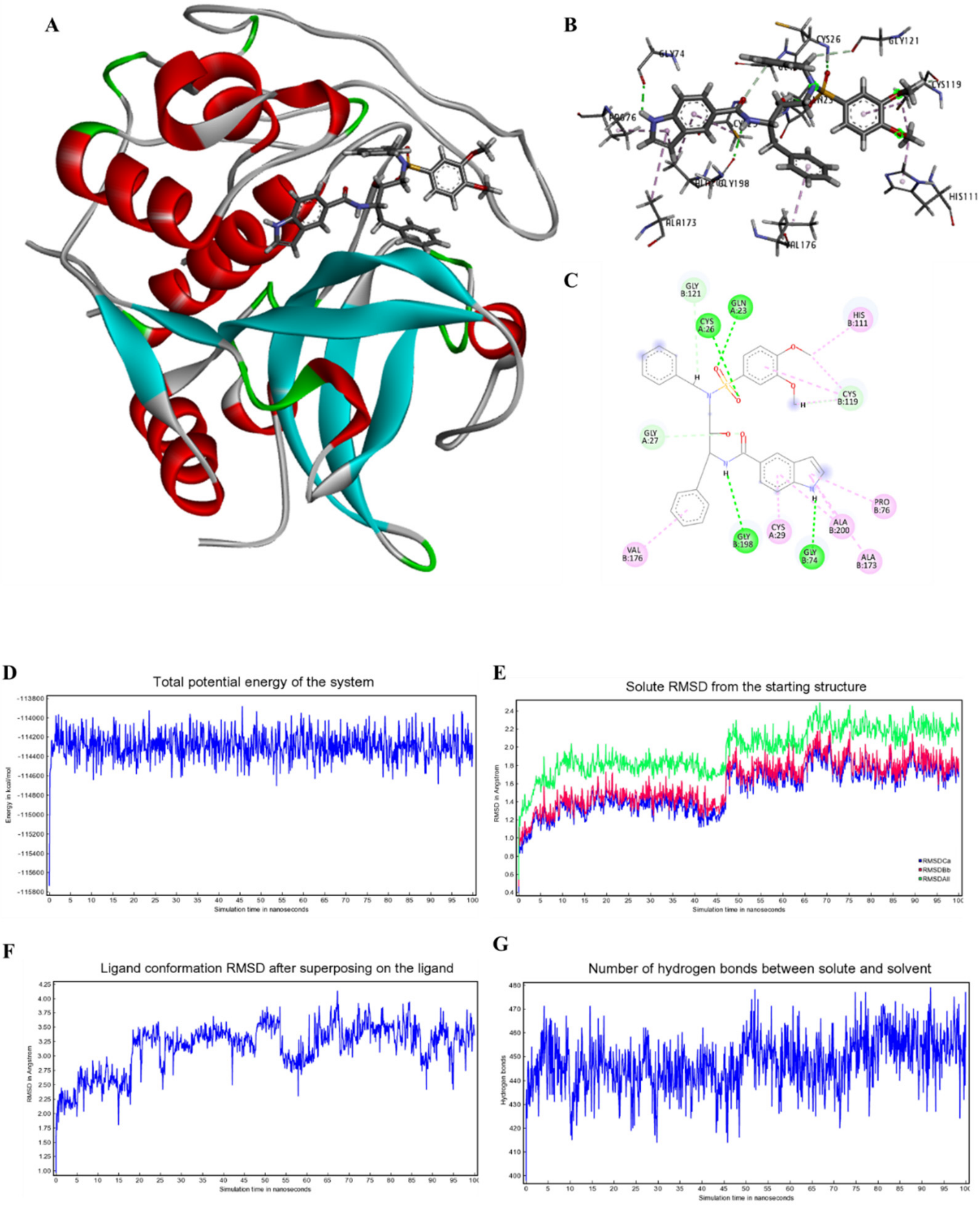

Figure S3. (**A**) Ribbon model of the best-docked poses for CatB/IBnDM. (**B**) Interaction profile of the docked poses of IBnDM and (**C**) 2D diagram interaction profile. (**D**) Total potential energy of the system. (**E**) Solute RMSD from the starting structure inside the binding pocket of the CatB enzyme. (**F**) Ligand conformation RMSD after superposing on the ligand. (**G**) Number of hydrogen bonds between solute and solvent of CatB enzyme.

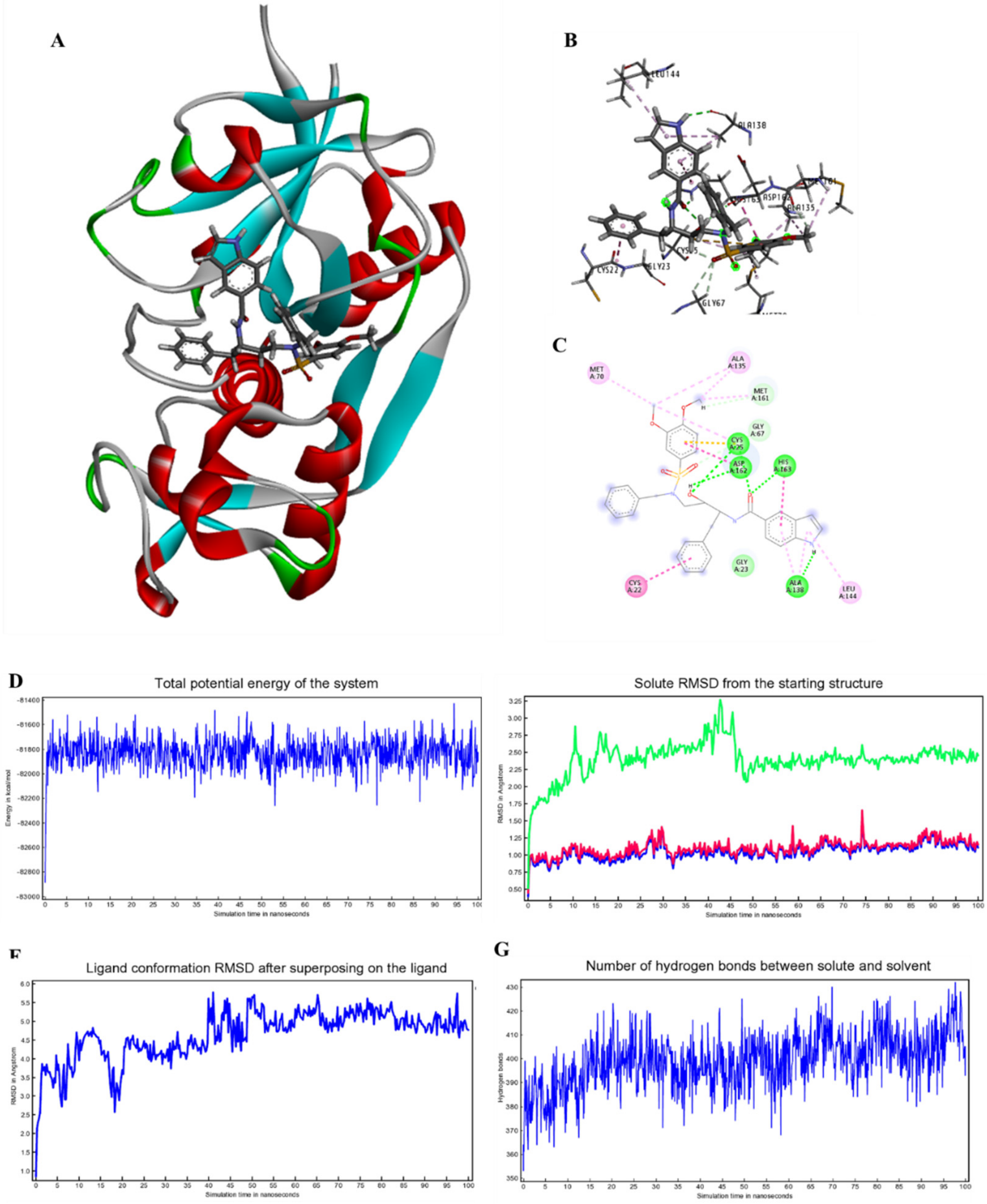

Figure S4. (**A**) Ribbon model of the best-docked poses for CatL/IBnDM. (**B**) Interaction profile of the docked poses of IBnDM and (**C**) 2D diagram interaction profile. (**D**) Total potential energy of the system. (**E**) Solute RMSD from the starting structure inside the binding pocket of the CatB enzyme. (**F**) Ligand conformation RMSD after superposing on the ligand. (**G**) Number of hydrogen bonds between solute and solvent of CatL enzyme.

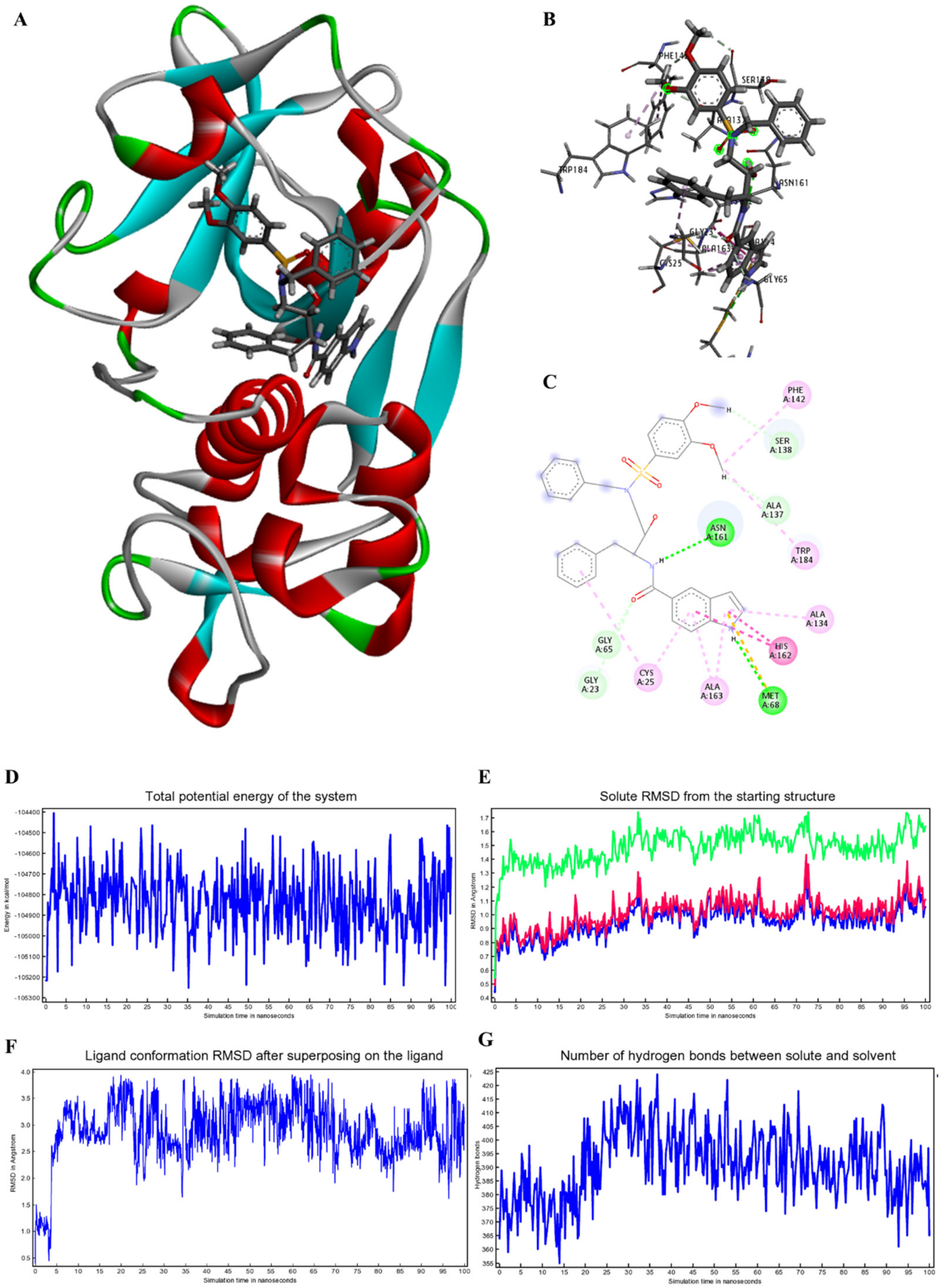

Figure S5. (**A**) Ribbon model of the best-docked poses for CatK/IBnDM. (**B**) Interaction profile of the docked poses of IBnDM and (**C**) 2D diagram interaction profile. (**D**) Total potential energy of the system. (**E**) Solute RMSD from the starting structure inside the binding pocket of the CatB enzyme. (**F**) Ligand conformation RMSD after superposing on the ligand. (**G**) Number of hydrogen bonds between solute and solvent of CatK enzyme.

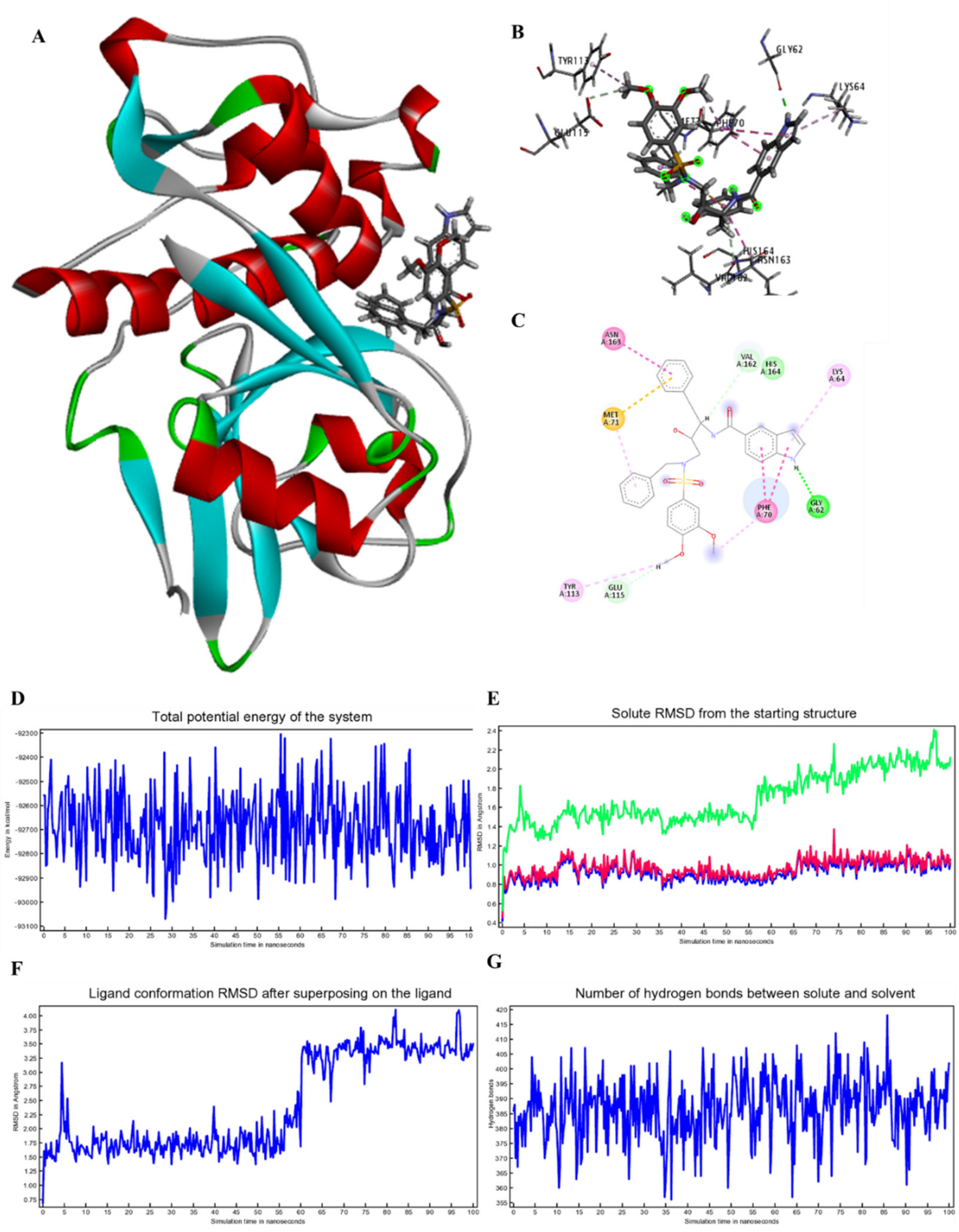

Figure S6. (**A**) Ribbon model of the best-docked poses for CatS/IBnDM. (**B**) Interaction profile of the docked poses of IBnDM and (**C**) 2D diagram interaction profile. (**D**) Total potential energy of the system. (**E**) Solute RMSD from the starting structure inside the binding pocket of the CatB enzyme. (**F**) Ligand conformation RMSD after superposing on the ligand. (**G**) Number of hydrogen bonds between solute and solvent of CatS enzyme.

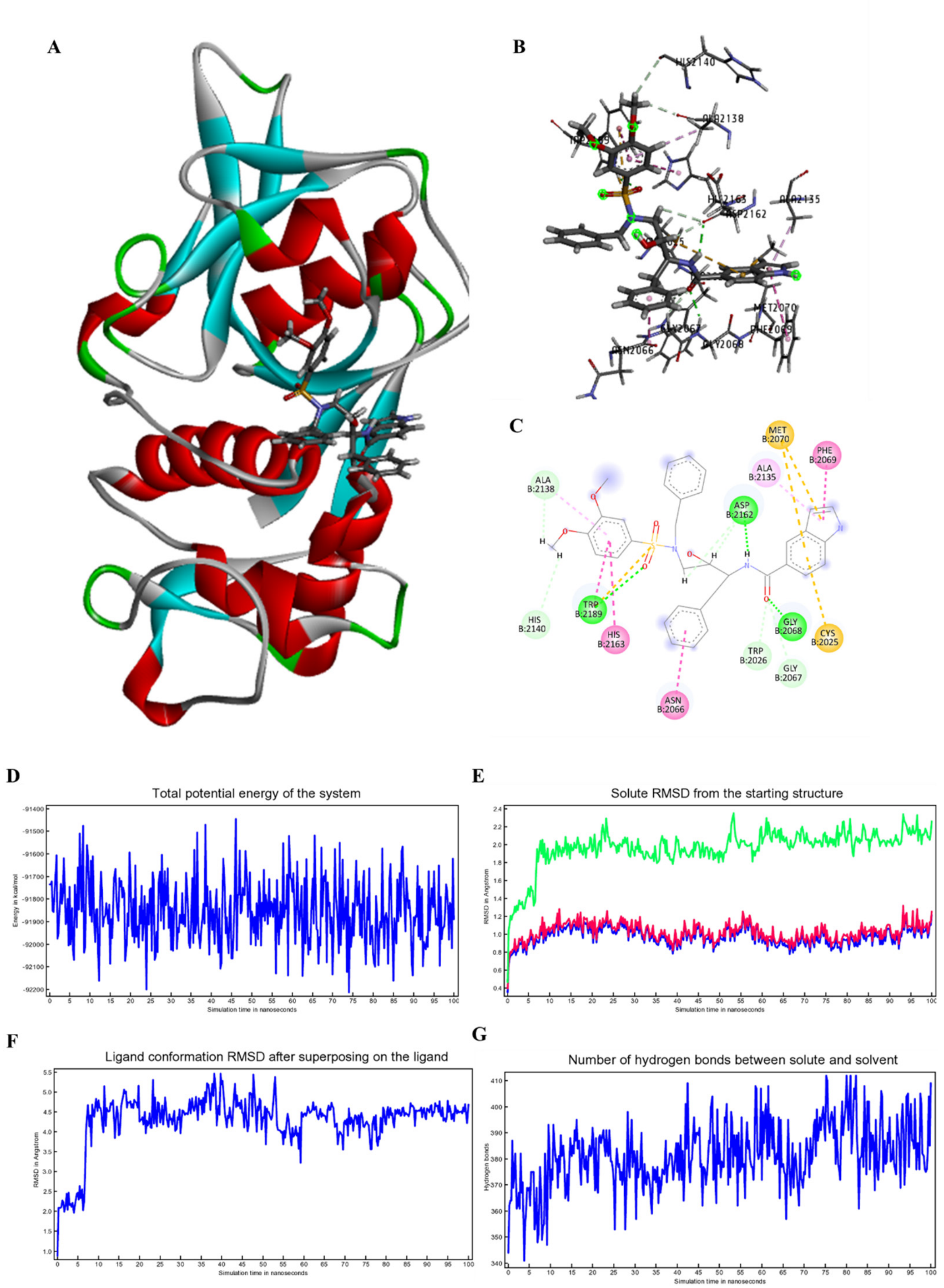

Figure S7. (**A**) Ribbon model of the best-docked poses for CatV/IBnDM. (**B**) Interaction profile of the docked poses of IBnDM and (**C**) 2D diagram interaction profile. (**D**) Total potential energy of the system. (**E**) Solute RMSD from the starting structure inside the binding pocket of the CatB enzyme. (**F**) Ligand conformation RMSD after superposing on the ligand. (**G**) Number of hydrogen bonds between solute and solvent of CatV enzyme.

Table S4. Half maximal inhibitory concentration (IC_{50} mM) values of compounds *in vitro* test.

Compound	$3CL^{pro}$	PL^{pro}
IBuDM	0.45	0.75
IBnDM	0.243	0.43
BTBnDM	0.427	0.17
BFBnDM	0.099	0.28

Cytotoxicity assay

The toxic effect of screened compounds was investigated following exposure to different concentrations of IBuDM, IBnDM, BTBnDM, and BFBnDM for 24 h, 48 h, and 72 h (Figure S8). The 50% cytotoxic concentration (CC_{50}) was reported in Table S5.

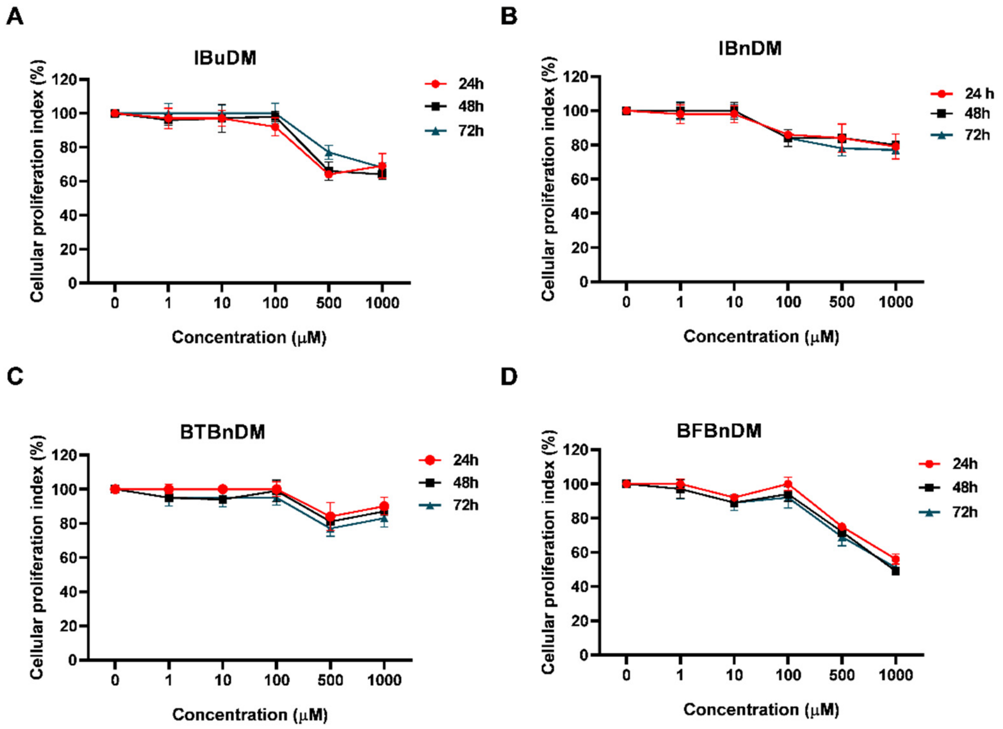

Figure S8. Cytotoxicity assayin Vero cells in response to A) IBuDM, B) IBnDM, C) BTBnDM, and D) BFBnDM. Vero cells were seeded at the density of 20,000 cells/well in a 96-well plate containing 100 μL complete media. For adherence, the cells were incubated for 24 h at 37 °C in a humidified 5% CO_2 incubator. After 24 h incubation, the media was replaced with fresh media, and Vero cells were treated with the four compounds at concentrations ranging between 1 to 1000 μM. Untreated cells were considered as a negative control, and DMSO-treated cells were vehicle controls. After the treatment, the cells were incubated at 37 °C in a humidified 5% CO_2 incubator. 24 h, 48 h, and 72 h hour posttreatment, 10 μL of WST-8 Solution. The absorbance was measured at 460 nm using a GLOMAX plate reader.

Table S5. Half maximal cytotoxic concentration (CC_{50} mM) values of studied compounds on VERO cells.

Compound	24 h	48 h	72 h
IBuDM	2.2	1.6	1.8
IBnDM	87	107	22
BTBnDM	40	19	19
BFBnDM	1.1	1	1.1

Production and characterization of pseudotype virus-like particles

To study SARS-CoV-2 infection in a BSL-2 laboratory, α-SARS-CoV-2 pseudovirus particle production and infection system was constructed by using a lentiviral vector bearing luciferase gene reporter for easy observation and analysis. The protocol to generate SARS-CoV-2 pseudoviruses was reported in Materials and Methods. Figure S2, panel A, shows the graphical representation of the plasmids' co-transfection strategy in HEK-293T. The production efficiency of the pseudoviruses and the titer was assessed by measuring the luciferase activity 72 h after pseudovirus inoculum transduction by Luciferase Assay System (Promega) according to the manufacturer's instructions. The results report the high infectivity of both S-incorporated pseudoviruses.

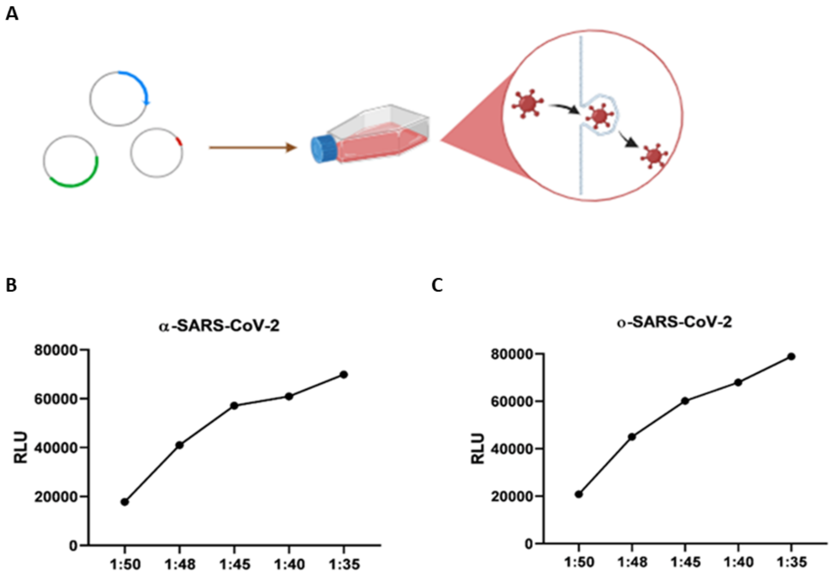

Figure S9. Production and Titration of pseudotype virus-like particles. A) graphical representation of pseudotype virus-like particle production. HEK293T cells were inoculated in a T75 flask and subjected to a three-plasmids co-transfection strategy to produce pseudotype virus-like particles bearing alpha and omicron spike protein. B and C) The titer of the α-SARS-CoV-2 pseudovirus and o-SARS-CoV-2 pseudovirus was measured by quantification of the luciferase activity. Vero cells were seeded in 96-well plates at 50–60% confluency. Then, the media was removed from each well and replaced with 50 µL of the serially diluted pseudotyped virus in DMEM with 10% FBS. The wells without the addition of the pseudovirus served as control cells. After 2 h of incubation in a 5% CO_2 environment at 37 °C, the pseudovirus inoculum was substituted with growth medium for 72 h. The infectivity assay was assessed by measuring the luciferase gene expression by using the Luciferase Assay System (Promega) according to the manufacturer's instructions.
